# Feasibility, Usability, and Acceptability of an Adaptive Mobile Health Medication Adherence Intervention for Youth: Iterative Mixed Methods Human-Centered Design Study

**DOI:** 10.2196/87124

**Published:** 2026-08-05

**Authors:** Caitlin S Sayegh, Shinyi Wu, Stephanie Lopez, Aridenne Dews, Jay Fleming, Daniela Cortez, Jan Portillo, Tamara Menendez, Marvin Belzer, Amy E West

**Affiliations:** 1 Department of Pediatrics, Keck School of Medicine, University of Southern California, Los Angeles, CA, United States; 2 Division of Adolescent and Young Adult Medicine, Children's Hospital Los Angeles, 4650 Sunset Blvd, MS#2, Los Angeles, CA, 90027, United States, 1 3233617748; 3 Suzanne Dworak-Peck School of Social Work, University of Southern California, Los Angeles, CA, United States; 4 Viterbi School of Engineering, University of Southern California, Los Angeles, CA, United States; 5 The Saban Research Institute, Children's Hospital Los Angeles, Los Angeles, CA, United States; 6 Department of Psychology, University of Southern California, Los Angeles, CA, United States; 7 Community Advisory Board, Children's Hospital Los Angeles, Los Angeles, CA, United States; 8 Department of Psychiatry and the Behavioral Sciences, University of Southern California, Los Angeles, CA, United States; 9 Department of Psychology, Children's Hospital Los Angeles, Los Angeles, CA, United States

**Keywords:** adolescents, young adults, mobile health, adherence, human-centered design, community advisory board, chronic health conditions

## Abstract

**Background:**

Adolescents and young adults with chronic health conditions often struggle to adhere to their daily oral medications. Transdiagnostic mobile health (mHealth) interventions have the potential to promote medication adherence by reaching youth at a large scale.

**Objective:**

This study aimed at designing an adaptive medication adherence mHealth intervention (Adaptive Cell Phone Support), guided by iterative feasibility, usability, and acceptability feedback. A secondary objective was to explore changes in self-reported medication adherence during a field trial.

**Methods:**

Using human-centered design methods, researchers collaborated with a community advisory board of young adult patients to conduct 3 cycles of iterative design and usability testing. Adolescents and young adults aged 15‐20 years (N=22) were recruited from a large pediatric hospital to user-test the intervention. Data collection included self-report questionnaires, think-aloud usability testing, semistructured interviews, and a 3-week field trial. Quantitative measures included the mHealth App Usability Questionnaire Ease of Use and Usefulness subscales, the Theoretical Framework of Acceptability Questionnaire, and visual analogue scales assessing medication adherence, as well as enrollment and engagement metrics. Qualitative data were analyzed using rapid assessment methods to identify actionable design insights, while quantitative data were analyzed using descriptive statistics and paired-samples *t* tests with a Holm-Bonferroni correction.

**Results:**

Enrollment was 63% and participants completed a mean of 67.2% (SD 23.5) of automated check-ins. Usability and acceptability ratings were relatively high across prototypes (eg, mHealth App Usability Questionnaire Ease of Use was mean 6.40, SD 0.64 for the initial prototype and mean 6.31, SD 0.61 for the third prototype, on a 7-point scale; Theoretical Framework of Acceptability was mean 4.33, SD 0.52 for the initial prototype and mean 4.50, SD 0.53 for the third prototype, on a 5-point scale). Qualitative data emphasized that the intervention was simple, easy to use, convenient, appropriate, and helpful for staying accountable for medication adherence, while also highlighting areas for improvement. Uncontrolled, 2-tailed, pre-post *t* tests estimated medium-sized improvements in self-reported medication adherence. However, only the percentage of time taking medications over the past month significantly increased (*t*_21_=3.26, *d*=0.70, 95% CI 0.22-1.16; *P*=.004).

**Conclusions:**

Integrated qualitative and quantitative results still suggest that more refinement is needed to optimize the intervention. Partnering with community members early in the development of an intervention may improve the ultimate feasibility, usability, and acceptability of digital health tools. Human-centered design offers a rapid, practical, and creative framework for identifying what works and what needs to be improved early in the lifecycle of a new intervention.

## Introduction

### Background

Approximately 25% of adolescents and young adults have chronic health conditions [1]. Many are prescribed daily oral medications. Yet, nonadherence rates commonly exceed 50% [2], contributing to poorer health outcomes and, for some conditions, higher mortality risk [3]. Given prevalent cell phone use among adolescents and young adults [4], mobile health (mHealth) interventions represent a promising, scalable approach to supporting medication adherence [5]. mHealth tools incorporating human support are often more effective than fully automated systems, consistent with the Supportive Accountability Model, which posits that accountability to a supportive human can enhance engagement in health behaviors [6,7]. Furthermore, integrating human support into mHealth interventions may reduce health disparities [8,9].

There are several promising interventions consistent with the Supportive Accountability Model. One is E-VOLUTION, a 2-way SMS text messaging intervention offering support from case managers to youth living with HIV [10]. E-VOLUTION participants who texted most with case managers showed the greatest medical appointment adherence and viral suppression [10]. Another intervention guided by the Supportive Accountability Model is Cell Phone Support, a phone call intervention offering brief, frequent problem-solving support to help adolescents and young adults overcome adherence barriers. In a randomized controlled trial, Cell Phone Support helped youth living with HIV improve medication adherence and psychosocial outcomes and reduce viral load [11,12]. Additionally, Teens Taking Charge, an online self-management intervention augmented by calls from coaches, has demonstrated efficacy for reducing pain and improving quality of life among adolescents and young adults with juvenile idiopathic arthritis [13] and increasing knowledge, self-efficacy, and transition preparedness among adolescents and young adults with hemophilia [14].

A pervasive barrier to reaching adolescents and young adults with chronic health conditions (CHC) at scale has been the tendency to design interventions specific to certain diagnoses (eg, HIV and diabetes) [15,16]. Adherence barriers are similar across conditions [17,18], and change mechanisms can promote adherence across diagnoses (eg, problem-solving skills [19-21], habit formation [22,23], and self-efficacy [24-26]). Yet, surface-level aspects of intervention design (eg, names, images referencing specific diagnoses, or medications) or study characteristics (eg, researchers enrolling from a population with a single diagnosis and funders limiting investigations to particular diseases) make it challenging to apply mHealth interventions broadly to adolescents and young adults with CHC. There are compelling justifications for focusing mHealth interventions on specific diagnoses, such as targeting the unique aspects of treatment regimens, enhancing community belongingness within a condition, and minimizing sample and outcome measure heterogeneity for scientific control and analytic precision. However, there is a notable gap in mHealth research targeting a behavior (eg, medication adherence) rather than a diagnosis—a strategy that could produce highly scalable interventions applicable to many adolescents and young adults, including those with multiple or rare conditions.

At the same time, technological advances have increased the capacity to adapt an intervention to the specifics of an individual, rather than creating and testing more static interventions population-by-population [27]. Adaptive digital health interventions are promising for supporting illness self-management by tailoring content and support to individual needs across diverse populations [28,29]. Studies show that users, including adolescents, value tailored mHealth approaches and report satisfaction with adaptive interventions [30]. Balderas-Díaz and colleagues [31] provide a helpful model for attending to usability and acceptability of adaptive digital health interventions early in the design process. Human-centered design (HCD) methods offer creative, repeatable procedures for building interventions that are desirable to humans, technologically feasible, and practically viable [32]. By centering the user experience, HCD can strengthen the translation potential of interventions [33-36], which is particularly important when designing youth-friendly interventions [37].

### Rationale and Objectives of This Study

Our team identified Cell Phone Support as highly scalable because it offers remote, time-limited support from paraprofessionals and applies broadly applicable mechanisms such as supportive accountability and problem-solving [11]. However, our prior research indicated a need to increase prospective acceptability (details in Multimedia Appendix 1). With guidance from our community advisory board (CAB) of young adults with CHC, we decided to redesign the program using a “foot-in-the-door” approach, a strategy where a small, initial request increases the likelihood of engaging with a larger request later [38,39]. Together, we planned an adaptive model beginning with low-intensity automated coaching and adding human coaching only when needed. We anticipated that this format would also enhance scalability by reducing workforce demands.

The goal of this study was to redesign Cell Phone Support into Adaptive Cell Phone Support, using iterative HCD cycles in collaboration with a CAB [32-36]. The primary objective was to use iterative mixed methods user testing to enhance the intervention’s feasibility, usability, and acceptability. An exploratory aim was to describe pre-post changes in self-reported medication adherence during a field trial. This study addresses 2 critical gaps in the adolescent and young adult adherence promotion literature. First, few transdiagnostic mHealth interventions targeting medication adherence have been developed. Second, there has been limited use of participatory HCD to optimize adherence interventions for adolescents and young adults.

## Methods

### Sample

The sample included 22 adolescents and young adults receiving subspecialty care at Children’s Hospital Los Angeles (CHLA), across 3 sequential user-testing cohorts as intervention prototypes were refined. Cohort 1 included 6 participants (January-May 2024), cohort 2 included 6 participants (October-December 2024), and cohort 3 included 10 participants (January-February 2025). Inclusion criteria were (1) ages of 15‐20 years, (2) hospital patient, (3) taking daily oral medication, (4) smartphone access, (5) English speaking, and (6) sufficient cognitive capacity to understand study procedures. We recruited a purposive sample, with various genders, ethnicities, CHC, medication regimens, and adherence barriers.

### Ethical Considerations

We obtained informed consent, parental or legal guardian permission, and assent from all participants according to their age, following procedures overseen by the CHLA Institutional Review Board (CHLA-23‐00149). Recruitment and consent conferences were held via hospital-approved teleconferencing platforms or in-person, in private locations. Patients, parents, and legal guardians were provided a copy of the consent form and time to consider their decision. Participants received US $80. In preparing this manuscript, participants’ confidentiality was protected by deidentifying data.

### Study Design

This study involved formative research to develop an intervention (NCT05719064). The study included iterative HCD and usability testing methods, pursued in partnership with a CAB.

### Intervention Prototype Co-Design Procedures

To redesign Cell Phone Support into Adaptive Cell Phone Support, we used participatory co-design and HCD because these approaches support the development of interventions that reflect end users’ needs [40-42]. We partnered with the CAB throughout the design process (July 2023 to March 2025; see Multimedia Appendix 2 for meeting agendas and resulting action items). We started by creating the initial intervention prototype, using the Computerized Intervention Authoring System (CIAS) [43]. CIAS is designed for behavioral scientists to easily prototype and evaluate digital interventions, without needing advanced technical skills. Meetings followed semistructured agendas with “make” (eg, prompting CAB members to draw or create visual representations of their intervention ideas), “tell” (eg, asking members to verbally express narratives and preferences relevant to the intervention), and “enact” prompts (eg, supporting adolescents and young adults in trying prototypes built using the CIAS platform) [44,45]. After 3 meetings, we reached consensus that the prototype was ready for user testing. Then, we held additional meetings after each iterative user-testing cohort to collaboratively interpret deidentified data summaries, make changes to the intervention design, and plan for dissemination and future research. CAB members received US $40 gift cards per meeting.

### Intervention Description

**Figure 1.**
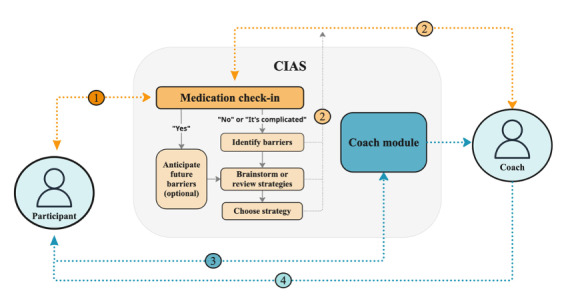
Adaptive Cell Phone Support system architecture. Numbers in the figure correspond to the following: (1) Participant receives adherence check-in SMS text invitation from CIAS based on preferred messaging frequency. Participant completes check-in. (2) Human coaching is added based on decision rules. (3) Participant can also request coaching via selecting the Coach module. (4) Coach reaches out to participant via their preferred contact method. CIAS: Computerized Intervention Authoring System.

Overall, Adaptive Cell Phone Support was designed to send SMS text messages to youth via CIAS asking them to click a link to open the automated coaching intervention on a web page. Then, youth were guided to check in about their current medication adherence; identify past, current, or future adherence barriers; brainstorm solutions; set an intention to try a strategy; and access resources or reach out to a coach for help. For example, one day a participant might indicate that they missed their medication because they were busy in band practice. They would then be prompted to generate a list of possible solutions on their own or select from a library of commonly used strategies (eg, setting an alarm and storing a backup dose). Next, they would be instructed to select a strategy to try. CIAS would then send an automated message within a few days to assess whether they were successful in taking their medication using the selected strategy, or whether they needed additional support to identify a different solution. The principal investigator would responsively add human coaching to a participant’s intervention plan based on decision rules (ie, not responding to 2 check-ins over 1 week, reporting 2 missed medication doses in 1 week, entering concerning text in open-text prompts, and requesting a coach). If coaching was indicated, a trained undergraduate volunteer contacted the participant to scaffold their problem-solving and provide supportive accountability as in the original Cell Phone Support model [11,46,47]. The coach also encouraged the participant to continue using the automated components. Participants selected the time of day to receive the check-in SMS text message invitations, which used varied greeting language and aimed for discretion (eg, “Hi! Use the link below to access today’s check-in.”). Most pages on the CIAS web page modules included an illustration from a free, online image library [48]. See Figure 1 for the system architecture diagram of Adaptive Cell Phone Support (Multimedia Appendix 3 for JavaScript).

### User-Testing Procedures

We applied convergent mixed methods to identify design adjustments needed to enhance feasibility, usability, and acceptability across 3 iterative user-testing cohorts. Participants were asked to complete (1) pretreatment self-report questionnaires, (2) a user-testing session with a think-aloud protocol [49] and open-ended interview questions, (3) immediate post–user-testing self-report questionnaires, (4) a 3-week intervention field test, (5) an exit interview, and (6) post–field test self-report questionnaires. Quantitative surveys were conducted via REDCap (Research Electronic Data Capture) [50]. Interviews were conducted by CS or SL via videoconferencing.

### Measures

Participants reported their demographic and personal characteristics on a survey administered after the user-testing session. We collected participants’ racial or ethnic self-reported identity to contextualize the sample, given structural factors shape inequitable access to care and digital health literacy. We abstracted electronic health record data to calculate the Medication Regimen Complexity Index [51].

The primary constructs investigated were feasibility, usability, and acceptability. Feasibility was characterized by enrollment rates and CIAS engagement metrics (eg, the frequencies with which participants accessed CIAS check-ins, completed problem-solving modules, and engaged with human coaching), similar to comparable formative intervention design studies [52,53]. In addition, we explored feasibility by having research personnel and CAB members test the intervention on their own devices ahead of enrolling participants and observed how intervention features were delivered in think-aloud testing.

Usability was assessed via qualitative data elicited in a think-aloud protocol and by asking participants to discuss the intervention’s understandability, learnability, operability, and attractiveness. The think-aloud protocol asked participants to verbalize their reactions, feelings, and thought process as they first interacted with the prototype. They were asked to use the program freely and then prompted to engage with the check-in module, problem-solving module, coach request module, and resource library, and narrate their inner thoughts and reactions. Usability was also assessed quantitatively using the mHealth App Usability Questionnaire (MAUQ) Ease of Use and Usefulness subscales [54]. The MAUQ shows strong reliability (α=.92) and criterion validity with adolescents and young adults [55]. Participants rated usability immediately after the think-aloud testing and again after the 3-week field testing.

Acceptability was measured quantitatively with the Theoretical Framework of Acceptability (TFA) Questionnaire [56] and by asking participants for a rating of the intervention from 1 to 5 stars [57]. The TFA Questionnaire is a standardized self-report scale, developed with prevalidation methods that obtained researcher and patient feedback on theory-derived items. Reliability has been fair to good on this measure (intraclass correlation coefficient=0.47‐0.70) [58]. Acceptability ratings were collected before participants used the intervention (prospective acceptability), after user testing, and after field testing. Acceptability was also assessed qualitatively via semistructured interviews guided by TFA constructs of affective attitude, burden, ethicality, intervention coherence, opportunity costs, perceived effectiveness, and self-efficacy [59], conducted immediately after user testing and again after field testing (guides in Multimedia Appendix 4).

Finally, to explore pre-post field-testing changes in medication adherence, participants completed a visual analogue scale (VAS) [60]. VAS asks participants to estimate the percentage of time out of 100% that they took their medications, took all their doses, and took their medications according to instructions over the past week and past month. VAS is simple to understand, shows convergent validity with behavioral observations of medication adherence, and, when administered via computer, is less influenced by response biases than other measures [61].

### Analysis

For quantitative data, summary statistics for feasibility, usability, and acceptability were calculated and inspected with the CAB after each cohort. Both item-level responses and total scores were reviewed to process user feedback and inform iterative intervention improvements. Missing data were not analyzed—only 2 questionnaire items were skipped by a single participant (n=1). Uncontrolled, pre-post changes in self-reported adherence were explored via paired-samples, 2-tailed *t* tests. Cohen *d* effect sizes were reported, and *P* values were interpreted after Holm-Bonferroni correction to control for the family-wise error rate.

Qualitative data were analyzed using rapid assessment [62-64]. Rapid assessment is well suited for processing targeted feedback on feasibility, usability, and acceptability to inform swift, iterative intervention development [65-67]. Qualitative responses were audio-recorded, summarized on templates (Multimedia Appendix 5), and transformed into data matrices. Either CS or SL completed each template based on interviews they had conducted. CS, SL, AD, and SW reviewed the qualitative data matrices and quantitative tables in 2-hour meetings after each cohort to prepare CAB meeting agendas and then discussed deidentified summaries with the CAB to identify actionable feedback to guide design changes. Through team discussions and CAB meetings, qualitative and quantitative data were integrated to reach consensus about which aspects of intervention prototypes were working well and which required improvement. Five undergraduate students also gave input at various analytic meetings that impacted integration of quantitative and qualitative results. The full results were shared with MB and AEW for review to strengthen interpretation during the writing process. Four CAB members (JF, DC, TM, and JP) also joined the writing process and were compensated with an additional US $40 gift card. We engaged in reflexivity, seeking to cultivate critical awareness of how our identities, goals, and assumptions influenced the research process and results [68].

## Results

### Participant Characteristics

Participants’ CHC included sickle cell disease, diabetes, HIV, cardiac conditions, cystic fibrosis, asthma, allergies, kidney disease, autoimmune disorders, gastroesophageal reflux disease, genetic conditions, pulmonary hypertension, and mental health conditions. Many had several co-occurring diagnoses. The sample was diverse in race or ethnicity, education, living situation, and employment (Table 1).

**Table 1.** Usability testing participant characteristics.

Variable	Summary statistic
Number of medications, mean (SD)	5.95 (3.76)
Medication Regimen Complexity Index, mean (SD*)*	21.66 (14.99)
Age (years), mean (SD)	18.09 (1.57)
Gender identity, n (%)	
Cisgender woman	14 (63.6)
Cisgender man	6 (27.3)
Transgender/nonbinary/other	2 (9.1)
Racial or ethnic identity, n (%)	
Latiné ethnicity	9 (40.9)
Black	4 (18.2)
White	5 (22.7)
Asian	2 (9.1)
American Indian/Alaska Native	0 (0.0)
Pacific Islander/Hawaiian Native	0 (0.0)
More than 1 race	7 (31.8)
Unknown/not reported	4 (18.2)
Living situation, n (%)	
Two biological parents	8 (36.4)
Biological mother only	5 (22.7)
Biological father only	1 (4.5)
Adoptive mother only	2 (9.1)
Adoptive father only	1 (4.5)
Other relatives	1 (4.5)
Roommates	3 (13.6)
Highest level of education*,* n (%)	
Graduated college or vocational school	1 (4.5)
Some college or vocational school	7 (31.8)
High school diploma or equivalent	4 (18.2)
12th grade	4 (18.2)
11th grade	4 (18.2)
10th grade	1 (4.5)
9th grade	1 (4.5)
Employed, n (%)	8 (36.4)

### Prototype Revisions Based on Integrated Feedback

After each cohort, we collaborated with the CAB to integrate qualitative and quantitative data to guide prototype revisions. The resulting design adjustments are detailed in Table 2.

**Table 2.** Iterative prototype revisions.

Prototype 1	Prototype 2	Prototype 3	Prototype 4 (For future research)
Design details			
Animated narrator feature guides CIAS^a^ check-in.	Remove animated narrator due to annoying quality, unexpected sound playing in public, and visual barrier to viewing content.	No change.	No change.
Program called “Adaptive Cell Phone Support” and described as an app.	No change.	Maintain “Adaptive Cell Phone Support” name but remove term “app” and describe as text messages and links to open web pages.	Maintain description but rename as one of these options: Building Blocks MyCoach MyHealthCoach HabitHelper
Sign-up module requests phone number and preferred time of day for messages.	No change.	Add orientation page with intervention description. Include image of a puzzle to illustrate problem-solving.	Maintain orientation page and add persuasive design social proof element [69] with promising data from usability testing cohorts.
Check-in language: “Did you take your medication today? (last required dose)”	No change.	Change check-in language: “Have you taken all of your prescribed medication since our last check-in?”	Add variety of greetings to reduce repetition, and open-ended question to show interest in youth beyond pill-taking.
Problem-solving module barrier options (each with 3 solution options): I forgot Medication issues It upsets me	No change.	Maintain existing. Add barriers: Hard to find privacy Left medicine at home No one reminded me ^·^ I’m trying to meet basic needs	Maintain existing. Add barriers: Schedule too busy I don’t think my medication helps Alter the barriers and solutions displayed over time based on prior selections.
Good-bye message: “See you next time! Thank you for checking in!” and thumbs up image	No change.	No change.	Add variety to goodbye language and images to reduce repetition.
Send resource library by SMS text message (list of phone numbers, apps, and websites) once a week.	No change.	Integrate resources into problem-solving module. Remove these weekly resource texts.	No change.
Send coaching invitation text once a week.	Maintain coaching invitation text. Add 30-second video of coaches explaining who they are and what coaching involves.	Maintain weekly text and video. Add live text from coach at initiation, midfield trial, and at the end of field trial.	Maintain all, plus add invitation for 5-minute phone call to get to know each other in first week to encourage use of coach if needed.
Offer human coaching when participants do not complete check-in or miss medication twice in a week, request coaching, or enter concerning open-text responses.	No change.	Maintain but if coaching is offered, encourage each participant to try a phone call if they are willing.	Maintain current approach and increase coaches’ references to what participants have input into the CIAS check-ins so that they do not feel they are repeating themselves.

a CIAS: Computerized Intervention Authoring System.

### Feasibility Results

Of the 35 participants screened eligible, 22 (63%) enrolled (ie, 6/12, 50% eligible in cohort 1; 6/8, 75% eligible in cohort 2; and 10/16, 63% eligible in cohort 3). At the beginning of the study, enrolling young cisgender men was challenging (ie, 0/5, 0% eligible cisgender men enrolled in cohort 1), but after recommendations from CAB members (eg, approaching eligible adolescents and young adults to ask for help building the intervention, rather than emphasizing how the intervention could help them) and encouraging clinicians to refer more young men, their enrollment improved (3/4, 71% eligible cisgender men enrolled in cohort 2; 3/3, 100% eligible cisgender men enrolled in cohort 3). Enrollment among eligible young women was initially high (6/7, 86% eligible cisgender women enrolled in cohort 1) but declined slightly over successive cohorts (2/3, 67% eligible cisgender women enrolled in cohort 2; 6/11, 55% eligible cisgender women enrolled in cohort 3). Three transgender or nonbinary youth were screened eligible for the study. Two enrolled (2/3, 67%). Only 4 participants declined participation outright; most who did not enroll never responded to outreach after completing a form to learn more about the study or being referred by their clinician. Three adolescents and young adults expressed that they were not interested, and 1 preferred to improve adherence on their own.

To characterize feasibility, we calculated the rates of participants completing different aspects of the intervention (Table 3). Almost all participants completed at least 1 check-in and 1 problem-solving module, with overall check-in adherence ranging from 59% to 76% across cohorts. Coaching was less often used. We did not observe clear trends of improvement in feasibility over the successive cohorts. We found no evidence of an intervention feature not being delivered as intended via research personnel and CAB member examination or observation during think-aloud testing.

**Table 3.** Intervention engagement as indicators of feasibility.

	Cohort 1 (n=6)	Cohort 2 (n=6)	Cohort 3 (n=10)
Engagement metrics			
Ever completed a check-in, n (%)	6 (100)	6 (100)	9 (90)
Check-ins completed, mean (SD)	70.8% (26.26)	59.9% (24.12)	75.8% (24.05)
Ever used problem-solving, n (%)	6 (83)	4 (67)	9 (90)
Ever requested coaching, n (%)	3 (50)	5 (83)	2 (20)
Ever triggered coaching, n (%)	4 (67)	4 (67)	7 (70)
Ever engaged with coach, n (%)	4 (67)	5 (50)	6 (60)

### Usability Results

Across all 3 cohorts, qualitative usability themes characterized the CIAS components of the intervention as “simple and easy to use,” “quick,” “smooth,” and “convenient.” For example, a participant with chronic pain explained: “It wasn’t hard...self-explanatory.” Another participant with a genetic condition commented: “Super simple...straightforward.”

Participants also indicated usefulness, reporting it “makes you think,” which helped them achieve better medication adherence. For example, a participant with cystic fibrosis and comorbid conditions explained:

*It brings awareness that you probably don’t usually have...Kind of putting it out there that you did forget a medication...It helps you kind of admit that. Sometimes I’ll miss a medication and I won’t really think too much about it...If I kind of sit and think, I’m like, “Ok, I forgot this one and this one*.”

Another participant with HIV stated: the intervention makes you “consciously think about what is it that’s stopping you from taking your meds.”

Despite participants finding the intervention mostly easy and useful, qualitative data from the think-aloud activity and interview questions highlighted usability barriers that informed prototype improvements. Participants found some intervention language imprecise or confusing and noted that common adherence barriers were missing from the bank. Others reported difficulty noticing all intervention components, particularly when multiple SMS text messages arrived at unexpected times or the animated narrator obscured text or images. The animated narrator also made the intervention less discreet. The absence of confirmation after completing check-ins left participants unsure whether they had finished it, occasionally causing them to repeat check-ins. Finally, some resource pages immediately triggered a phone call when clicked, resulting in confusion for participants who were not attempting to place a call but rather learn more about the resource. We noticed fewer usability barriers during user-testing sessions as the prototypes were iterated. Participants provided more constructive feedback regarding usability after the user-testing session than after the field trial. The most difficult period appeared to be the stage of learning and adopting the intervention. After trying it for 3 weeks, participants did not discover new usability problems and worked out most of any confusion they had during their initial intervention exposure.

Quantitative usability scores were near the top of the 7-point scale throughout the study and did not differ noticeably from the user-testing session or field trial (Table 4). Scores were not observed to rise over the iterative prototypes. Ease of use was rated higher than usefulness. Lower-item level scores highlighted specific usability concerns requiring attention in prototype iterations. Concerns included views on whether the intervention improved access to services, whether participants could be confident their information would be received, whether the intervention increased opportunities to interact with providers, and whether the intervention made it convenient to communicate with providers. Integrating these quantitative data with the qualitative data, these lower ratings could be related to confusion or doubts participants expressed in their interviews about the role of the coach in the intervention. For example, a few participants explained that the coaches were repeating the same questions or solutions that the CIAS platform had asked them, making them feel like their CIAS responses had not been reviewed by the coach. Others explained that they did not know what would happen if they asked to talk to the coach, so they never requested support.

**Table 4.** Usability and acceptability ratings.

	Cohort 1 (n=6)	Cohort 2 (n=6)	Cohort 3 (n=10)
Usability, mean (SD)			
MAUQ^a^ Ease of Use Subscale, immediate post–user testing (T1)	6.88 (0.58)	6.65 (0.46)	6.56 (0.45)
MAUQ Ease of Use Subscale, post–3-week field testing (T2)	6.40 (0.64)	6.29 (0.63)	6.31 (0.61)^b^
MAUQ Usefulness Subscale (T1)	6.50 (0.52)	5.71 (1.40)	5.68 (1.13)
MAUQ Usefulness Subscale (T2)	6.45 (0.54)	5.76 (1.31)	5.69 (1.12)
Acceptability, mean (SD)			
Stars out of 5, pre–user testing (T0)	4.25 (0.96)	4.50 (0.84)	4.20 (0.63)
Stars out of 5, immediate post–user testing (T1)	4.75 (0.50)	4.67 (0.52)	4.70 (0.48)
Stars out of 5, post–3-week field testing (T2)	4.80 (0.45)	4.50 (0.55)	4.10 (0.88)
TFA^c^ Global acceptability (T0)	4.33 (0.52)	3.33 (1.86)	4.50 (0.53)
TFA Global acceptability (T1)	4.67 (0.52)	4.83 (0.41)	4.70 (0.48)
TFA Global acceptability (T2)	4.50 (0.55)	4.33 (0.82)	4.50 (0.71)

a MAUQ: mHealth App Usability Questionnaire.

b One participant skipped 2 items, which contributed to a missing Ease of Use subscale for that participant. Item-level responses in Multimedia Appendix 6.

c TFA: Theoretical Framework of Acceptability.

Although participants did identify areas where the intervention’s usability could be enhanced, overall perceptions across qualitative and quantitative responses suggested that the intervention was relatively usable.

### Acceptability Results

Qualitative responses suggested that participants found the intervention relatively acceptable. Participants described the intervention positively because it kept them focused on taking medication, made them feel “accountable,” was “reassuring” and “soothing,” had a “motivational type of feel,” and was “judgment-free.” A participant with sickle cell disease explained: “I think the program is really good. So far, I feel like having the program has been like kind of having an accountability partner.” Another participant with HIV shared: “Now you have more of a reason [to take your medications] because you’re being checked on. It’s motivation.” These positive assessments were usually the first types of comments participants made in response to think-aloud prompts or open-ended questions. The qualities of these comments were similar at the user-testing session and after the field trial.

Many responses specifically evaluated the intervention images, the flexibility of time spent on each intervention activity, and the option of speaking to a human coach as enhancing acceptability. Many participants responded with positive affect, indicating that they enjoyed the intervention. They often used words such as “cute,” “cool,” or “fun” to describe the intervention. Only 1 person provided an affective response that was less positive, explaining that they felt “normal” while using the intervention. Participants did report that acceptability would depend on their current medication adherence; if they did not struggle with adherence, it might be “overkill.”

Some participants explained that immediately after learning about the intervention, acceptability could be low, but that after seeing and trying the intervention, they viewed the intervention as more acceptable. For example, a participant with pulmonary hypertension expected the intervention to be “generic” and “expressively bland, but a positive reinforcement kind of thing...” but they were surprised “it was always visually pleasing with a positive vibe and inviting human-to-human conversation concept.” A participant with HIV also described a negative initial view, giving way to a more positive assessment of the intervention, saying that they first thought, “The program could be very overwhelming because it’s a reminder of what you’re sick of...and you would have to talk about it.” However, after trying the intervention, they perceived it as “genuine” and “cheerful.”

Quantitative acceptability ratings for specific items showed some evidence of increasing across successive prototypes (Table 4). For example, in the first 2 cohorts, participants often indicated “no opinion” when asked whether the intervention seemed effective. By the third cohort, participants rated perceived effectiveness higher. Additionally, suboptimal ratings of intervention burden were given in the first cohort, and suboptimal opportunity costs were indicated in the second cohort—guiding design changes to streamline tasks. By the third cohort, these aspects of acceptability were all improved such that participants perceived that the intervention required only a little effort and did not interfere with their lives.

Integrated qualitative and quantitative data focused the team on making several prototype adjustments to make the experience less taxing and better communicate how the intervention was intended to help through an orientation module.

### Exploratory Pre-Post Changes in Self-Reported Adherence

Participants self-reported their medication adherence over the past week and over the past month out of 100%, before the field trial began and after the 3-week field trial (Table 5). There were no missing data on this measure at either timepoint. Across all items, medium-sized effect changes were estimated. However, with Holm-Bonferroni corrections, only the increase in past month adherence was statistically significant.

**Table 5.** Medication adherence reported via visual analogue scale.

Variable	Pre (n=22)	Post (n=22)	*T* test results, 2-sided
Past week adherence, mean (SD)			
Percentage of time took medications	81.05 (21.70)	87.82 (13.92)	*t*_21_=2.31, *P*=.031; *d*=0.49, 95% CI (0.04-0.93)
Percentage of time took all doses	77.64 (25.77)	86.41 (17.28)	*t*_21_=2.25, *P*=.035; *d*=0.48, 95% CI (0.03-0.92)
Percentage of time according to instructions	74.95 (27.47)	86.27 (21.69)	*t*_21_=2.44, *P*=.024; *d*=0.52, 95% CI (0.07-0.96)
Past month adherence, mean (SD)			
Percentage of time took medications	73.95 (23.46)	84.68 (17.28)	*t*_21_=3.26, *P*=.004*^a^; *d*=0.70, 95% CI (0.22-1.16)
Percentage of time took all doses	73.36 (25.00)	80.86 (20.13)	*t*_21_=2.46, *P*=.022; *d*=0.53, 95% CI (0.07-0.97)
Percentage of time according to instructions	72.77 (27.78)	85.05 (21.84)	*t*_21_=2.29, *P*=.032; *d*=0.49, 95% CI (0.04-0.89)

a Asterisk symbol indicates statistical significance using Holm-Bonferroni method-corrected *P*-value thresholds.

## Discussion

### Principal Results

Qualitative and quantitative feasibility, usability, and acceptability data helped guide design adjustments for an adaptive medication adherence intervention for adolescents and young adults with CHC. Participants’ feedback suggested that Adaptive Cell Phone Support was easy to use and provided a pleasant experience that adolescents and young adults described as supportive and helpful for encouraging reflection about how to improve adherence. Ongoing areas for improvement include increasing the enrollment rate of eligible participants, possibly by strengthening early impressions that Adaptive Cell Phone Support is enjoyable, useful, and effective. User-testing and field-testing methods were productive for identifying usability barriers and informing design changes. Self-reported adherence appeared to improve during a 3-week field trial, though the small sample and lack of control group limit confidence in these preliminary results.

We expected to achieve stepwise improvement in feasibility, usability, and acceptability measures from cohort to cohort, based on iterative improvements to the intervention design. While the qualitative data did suggest reduced confusion over time after usability barriers were addressed in iterative prototypes, quantitative usability scores were similar across prototypes. The lack of quantitative changes could have several explanations. It could be that the design changes were insufficient to make a meaningful difference in adolescents’ and young adults’ perceptions of the intervention. In addition, there may have been a ceiling effect where participants already found the intervention highly usable and acceptable in the first prototype, leaving little room for improvement in subsequent prototypes. It is also possible that the cohorts differed in meaningful ways that made it less appropriate to compare their ratings. The first cohort heavily comprised young women enrolled in college, our team was more successful at recruiting young men in later cohorts, and we did not enroll those aged 15 or 16 years until the third cohort, which may have influenced the qualitative and quantitative results of each cohort unrelated to the prototype revisions. A strength of this study was the use of mixed methods measurement of usability and acceptability, through some of the best-validated questionnaires on these constructs and qualitative think-aloud and interview methods. Although the quantitative scores did not show clear patterns of change over the study, using theoretically grounded surveys ensured that our team paid attention to critical components of usability and acceptability.

HCD has only recently been applied within adolescent and young adult mHealth research; however, several published examples demonstrate how engaging adolescents and young adults in HCD may result in relevant, acceptable interventions [36,70,71]. Partnering with a CAB over 5 years (2020‐2025) helped to build trust, experience, and reciprocity between researchers and patient advisors. The long-term collaboration was able to produce proposals for pilot research funding that were authentically cocreated and prototypes informed by the adolescents’ and young adults’ experiences of managing CHC. Sustainability in CAB-partnered research requires formal planning, transparency, and mutual discussions [72]. This study illustrates how HCD and prototyping are well aligned with multiyear CAB collaborations spanning multiple projects and funders.

This formative study highlights critical questions for mHealth intervention researchers to investigate. How can effective medication adherence interventions be packaged to appeal to adolescents and young adults who are not taking medications consistently? What type or dosage of digital or human support for medication adherence is necessary, tolerable, or appreciated by adolescents and young adults with CHC? Next steps for the Adaptive Cell Phone Support design process are to conduct usability testing of a fourth prototype with a larger cohort, engage experts in a heuristic usability evaluation [73], and work with hospital patient, caregiver, and clinician advisory councils to complete user-centered cognitive walk-throughs [74]. With this remaining round of feedback and design iteration, we plan to finalize the intervention and complete a randomized pilot trial to assess the preliminary efficacy of Adaptive Cell Phone Support.

### Comparison With Prior Work

In most ways, the redesigned Adaptive Cell Phone Support appeared feasible and usable for participants. To help contextualize these findings, we reviewed the results from our most recent Cell Phone Support study, which did not use an adaptive structure. Notably, the 2 studies differed substantially in design and population and were not harmonized for head-to-head comparison (eg, current field test lasted 3 weeks while prior pilot was 12 weeks; this study enrolled a wide range of diagnoses, while the prior study enrolled only those with solid organ transplants, sickle cell disease, or type 2 diabetes). Within this broader context, the Adaptive Cell Phone Support enrollment rate was within the range previously achieved in our earlier work (63% in this study vs 68% previously) [47]. MAUQ Ease of Use and Usefulness scores also fell within a similar range [47]. The Adaptive Cell Phone Support protocol did require less human coach effort (0.94 calls or texts per week per participant in Adaptive Cell Phone Support versus 3.83 calls or texts per week per participant in Cell Phone Support), and yet participants continued to describe the intervention as a motivating reminder where they felt cared for and accountable for better adherence, similar to the completely human-delivered version of Cell Phone Support [75]. Although this study was motivated by the idea that an adaptive format of Cell Phone Support might enhance acceptability, the formative findings did not support this conclusion. More intervention design attention is needed to increase eligible participants’ willingness to try Adaptive Cell Phone Support and enhance acceptability.

### Limitations

This study has several limitations. First, the study focused primarily on feasibility, usability, and acceptability, with the intent of informing iterative design improvements. No a priori benchmarks were defined to determine whether adequate thresholds were met, limiting conclusions about readiness for broader implementation. An additional limitation is that CIAS does not currently produce log-derived technical metrics for researchers to further characterize feasibility. Also, despite the benefits of long-standing CAB partnership, by the end of the process, CAB members were aged 23‐27 years, putting them in a different life stage than the younger adolescents in the current usability study.

Another weakness of the study is the lack of traditional qualitative analysis, which could have been more rigorous in defining qualitative themes from participants’ responses. However, the rapid assessment process allowed adequate depth and timely, efficient feedback to guide refinement of the intervention. Furthermore, although cohort sample sizes were selected based on models in the user-testing literature and were sufficient to generate actionable design feedback, we found it difficult to make conclusions about differences in feasibility, usability, or acceptability between cohorts over time due to the possibility that small samples represented idiosyncratic perspectives. Especially given the heterogeneity in ages and CHC represented, small groups led us to feel less certain we understood how adolescents and young adults more broadly would respond to the intervention. In addition, this study was not designed to evaluate efficacy. The exploration of increases in self-reported adherence is very preliminary. Future efficacy studies should use multiple measures of adherence for triangulation (eg, biochemically validated measures, pharmacy possession refill data, medication event monitoring, and ecological momentary assessments). Finally, this study was not designed to evaluate whether this transdiagnostic intervention is similarly feasible, usable, acceptable, or efficacious across demographic and medication regimen characteristics, or compare an adaptive format with a nonadaptive format.

### Conclusions

The study suggests that human-centered, CAB-guided design can produce mHealth adherence tools that are workable for youth with a wide spectrum of CHC and varying medication regimens. However, additional design efforts are needed to enhance prospective intervention acceptability.

## Supplementary material

10.2196/87124 Multimedia Appendix 1 Brief history of Cell Phone Support intervention and this team’s rationale for redesign.

10.2196/87124 Multimedia Appendix 2 Community Advisory Board agendas and resulting action items.

10.2196/87124 Multimedia Appendix 3 JSON underlying Computerized Intervention Authoring System intervention.

10.2196/87124 Multimedia Appendix 4 Think-aloud guide and semistructured interview guides.

10.2196/87124 Multimedia Appendix 5 Rapid assessment template.

10.2196/87124 Multimedia Appendix 6 Usability and acceptability item-level ratings.

## Abbreviations

CAB: community advisory board
CHC: chronic health conditions
CHLA: Children’s Hospital Los Angeles
CIAS: Computerized Intervention Authoring System
HCD: human-centered design
MAUQ: mHealth App Usability Questionnaire
mHealth: mobile health
REDCap: Research Electronic Data Capture
TFA: Theoretical Framework of Acceptability
VAS: visual analogue scale

## References

[1] (2021). Trends in prevalence of disabilities among youth. United States Department of Health and Human Services Office of the Assistant Secretary for Health Webpage.

[2] Rapoff MA, Duncan C, Karlson C (2023). Adherence to Pediatric Medical Regimens.

[3] Christensen AJ, Johnson JA (2002). Patient adherence with medical treatment regimens: an interactive approach. Curr Dir Psychol Sci.

[4] Anderson M, Faverio M, Gottfried J (2023). Teens, social media and technology 2023. Pew Research Center.

[5] Milat AJ, King L, Bauman AE, Redman S (2013). The concept of scalability: increasing the scale and potential adoption of health promotion interventions into policy and practice. Health Promot Int.

[6] Kim SK, Park SY, Hwang HR, Moon SH, Park JW (2025). Effectiveness of mobile health intervention in medication adherence: a systematic review and meta-analysis. J Med Syst.

[7] Mohr DC, Cuijpers P, Lehman K (2011). Supportive accountability: a model for providing human support to enhance adherence to eHealth interventions. J Med Internet Res.

[8] Gibbons MC, Fleisher L, Slamon RE, Bass S, Kandadai V, Beck JR (2011). Exploring the potential of Web 2.0 to address health disparities. J Health Commun.

[9] Arsenijevic J, Tummers L, Bosma N (2020). Adherence to electronic health tools among vulnerable groups: systematic literature review and meta-analysis. J Med Internet Res.

[10] Gerke DR, Glotfelty J, Schlueter J, Freshman M, Plax K (2020). E-VOLUTION: a text messaging-powered intervention-connection, support, and HIV eradication. Health Promot Pract.

[11] Belzer ME, Naar-King S, Olson J (2014). The use of cell phone support for non-adherent HIV-infected youth and young adults: an initial randomized and controlled intervention trial. AIDS Behav.

[12] Sayegh CS, MacDonell KK, Clark LF (2018). The impact of cell phone support on psychosocial outcomes for youth living with HIV nonadherent to antiretroviral therapy. AIDS Behav.

[13] Stinson JN, Lalloo C, Hundert AS (2020). Teens taking charge: a randomized controlled trial of a web-based self-management program with telephone support for adolescents with juvenile idiopathic arthritis. J Med Internet Res.

[14] Breakey VR, Ignas DM, Warias AV, White M, Blanchette VS, Stinson JN (2014). A pilot randomized control trial to evaluate the feasibility of an Internet-based self-management and transitional care program for youth with haemophilia. Haemophilia.

[15] Mulawa MI, LeGrand S, Hightow-Weidman LB (2018). eHealth to enhance treatment adherence among youth living with HIV. Curr HIV/AIDS Rep.

[16] Badawy SM, Barrera L, Sinno MG, Kaviany S, O’Dwyer LC, Kuhns LM (2017). Text messaging and mobile phone apps as interventions to improve adherence in adolescents with chronic health conditions: a systematic review. JMIR Mhealth Uhealth.

[17] Hanghøj S, Boisen KA (2014). Self-reported barriers to medication adherence among chronically ill adolescents: a systematic review. J Adolesc Health.

[18] Schwartz DD, Axelrad ME (2015). Healthcare Partnerships for Pediatric Adherence: Promoting Collaborative Management for Pediatric Chronic Illness Care.

[19] Gross R, Bellamy SL, Chapman J (2013). Managed problem solving for antiretroviral therapy adherence: a randomized trial. JAMA Intern Med.

[20] Hill-Briggs F, Lazo M, Peyrot M (2011). Effect of problem-solving-based diabetes self-management training on diabetes control in a low income patient sample. J Gen Intern Med.

[21] Greenley RN, Gumidyala AP, Nguyen E (2015). Can you teach a teen new tricks? problem solving skills training improves oral medication adherence in pediatric patients with inflammatory bowel disease participating in a randomized trial. Inflamm Bowel Dis.

[22] Badawy SM, Shah R, Beg U, Heneghan MB (2020). Habit strength, medication adherence, and habit-based mobile health interventions across chronic medical conditions: systematic review. J Med Internet Res.

[23] Eaton CK, Comer M, Pruette CS, Riekert KA (2023). Medication adherence in youths with CKD: habits for success. Pediatr Nephrol.

[24] Náfrádi L, Nakamoto K, Schulz PJ (2017). Is patient empowerment the key to promote adherence? A systematic review of the relationship between self-efficacy, health locus of control and medication adherence. PLoS One.

[25] Johnson MO, Neilands TB, Dilworth SE, Morin SF, Remien RH, Chesney MA (2007). The role of self-efficacy in HIV treatment adherence: validation of the HIV Treatment Adherence Self-Efficacy Scale (HIV-ASES). J Behav Med.

[26] Okuboyejo S, Mbarika V, Omoregbe N (2018). The effect of self-efficacy and outcome expectation on medication adherence behavior. J Public Health Afr.

[27] Nahum-Shani I, Qian M, Almirall D (2012). Experimental design and primary data analysis methods for comparing adaptive interventions. Psychol Methods.

[28] Setiawan IMA, Zhou L, Alfikri Z (2019). An adaptive mobile health system to support self-management for persons with chronic conditions and disabilities: usability and feasibility studies. JMIR Form Res.

[29] Spierling Bagsic SR, Savin KL, Soriano EC (2023). Process evaluation of Dulce Digital-Me: an adaptive mobile health (mHealth) intervention for underserved Hispanics with diabetes. Transl Behav Med.

[30] Cushing CC, Bejarano CM, Ortega A, Sayre N, Fedele DA, Smyth JM (2021). Adaptive mHealth intervention for adolescent physical activity promotion. J Pediatr Psychol.

[31] Balderas-Díaz S, Rodríguez-Fórtiz MJ, Garrido JL, Bellido-González M, Guerrero-Contreras G (2022). A psycho-educational intervention programme for parents with SGA foetuses supported by an adaptive mHealth system: design, proof of concept and usability assessment. BMC Med Inform Decis Mak.

[32] (2015). The field guide to human-centered design. Ideo.org.

[33] Lyon AR, Brewer SK, Areán PA (2020). Leveraging human-centered design to implement modern psychological science: return on an early investment. Am Psychol.

[34] Shrier LA, Burke PJ, Jonestrask C, Katz-Wise SL (2020). Applying systems thinking and human-centered design to development of intervention implementation strategies: an example from adolescent health research. J Public Health Res.

[35] Mukherjee TI, Zerbe A, Falcao J (2022). Human-centered design for public health innovation: codesigning a multicomponent intervention to support youth across the HIV care continuum in Mozambique. Glob Health Sci Pract.

[36] Stiles-Shields C, Cummings C, Montague E, Plevinsky JM, Psihogios AM, Williams KDA (2022). A call to action: using and extending human-centered design methodologies to improve mental and behavioral health equity. Front Digit Health.

[37] McCabe E, Amarbayan MM, Rabi S (2023). Youth engagement in mental health research: a systematic review. Health Expect.

[38] Guéguen N (2002). Foot-in-the-door technique and computer-mediated communication. Comput Human Behav.

[39] Freedman JL, Fraser SC (1966). Compliance without pressure: the foot-in-the-door technique. J Pers Soc Psychol.

[40] Bray EA, Everett B, George A, Salamonson Y, Ramjan LM (2022). Co-designed healthcare transition interventions for adolescents and young adults with chronic conditions: a scoping review. Disabil Rehabil.

[41] Thabrew H, Fleming T, Hetrick S, Merry S (2018). Co-design of eHealth interventions with children and young people. Front Psychiatry.

[42] Malloy J, Partridge SR, Kemper JA, Braakhuis A, Roy R (2023). Co-design of digital health interventions with young people: a scoping review. Digit Health.

[43] Ondersma SJ, Chase SK, Svikis DS, Schuster CR (2005). Computer-based brief motivational intervention for perinatal drug use. J Subst Abuse Treat.

[44] Vandekerckhove P, de Mul M, Bramer WM, de Bont AA (2020). Generative participatory design methodology to develop electronic health interventions: systematic literature review. J Med Internet Res.

[45] Harte R, Glynn L, Rodríguez-Molinero A (2017). A human-centered design methodology to enhance the usability, human factors, and user experience of connected health systems: a three-phase methodology. JMIR Hum Factors.

[46] Sayegh CS, Szmuszkovicz JR, Menteer J (2018). Cell phone support to improve medication adherence among solid organ transplant recipients. Pediatr Transplant.

[47] Sayegh CS, MacDonell KK, Iverson E (2024). Randomized pilot trial of cell phone support to improve medication adherence among adolescents and young adults with chronic health conditions. BMC Digital Health.

[48] (2024). Awesome free customizable illustrations for your next project. Storyset by the Freepik Company.

[49] Boren MT, Ramey J (2000). Thinking aloud: reconciling theory and practice. IEEE Trans Profess Commun.

[50] Harris PA, Taylor R, Minor BL (2019). The REDCap consortium: building an international community of software platform partners. J Biomed Inform.

[51] Hirsch JD, Metz KR, Hosokawa PW, Libby AM (2014). Validation of a patient-level medication regimen complexity index as a possible tool to identify patients for medication therapy management intervention. Pharmacotherapy.

[52] Adams LB, Watts T, DeVinney A (2024). Acceptability and feasibility of a smartphone-based real-time assessment of suicide among Black men: mixed methods pilot study. JMIR Form Res.

[53] McClure JB, Heffner JL, Krakauer C, Mun S, Catz SL (2024). A novel mHealth App for Smokers Living With HIV Who Are Ambivalent About Quitting Smoking: Formative Research and Randomized Feasibility Study. JMIR Form Res.

[54] Zhou L, Bao J, Setiawan IMA, Saptono A, Parmanto B (2019). The mHealth App Usability Questionnaire (MAUQ): development and validation study. JMIR Mhealth Uhealth.

[55] Connelly M, Boorigie M, McCabe K (2023). Acceptability and tolerability of extended reality relaxation training with and without wearable neurofeedback in pediatric migraine. Children (Basel).

[56] Sekhon M, Cartwright M, Francis JJ (2022). Development of a theory-informed questionnaire to assess the acceptability of healthcare interventions. BMC Health Serv Res.

[57] Perski O, Short CE (2021). Acceptability of digital health interventions: embracing the complexity. Transl Behav Med.

[58] Keyworth C, O’Connor R, Quinlivan L, Armitage CJ (2021). Acceptability of a brief web-based theory-based intervention to prevent and reduce self-harm: mixed methods evaluation. J Med Internet Res.

[59] Sekhon M, Cartwright M, Francis JJ (2017). Acceptability of healthcare interventions: an overview of reviews and development of a theoretical framework. BMC Health Serv Res.

[60] Walsh JC, Dalton M, Gazzard BG (1998). Adherence to combination antiretroviral therapy assessed by anonymous patient self-report. AIDS.

[61] Kalichman SC, Simbayi LC, Cloete A, Mthembu PP, Mkhonta RN, Ginindza T (2009). Measuring AIDS stigmas in people living with HIV/AIDS: the Internalized AIDS-Related Stigma Scale. AIDS Care.

[62] Palinkas LA, Mendon SJ, Hamilton AB (2019). Innovations in mixed methods evaluations. Annu Rev Public Health.

[63] Hamilton AB (2013). Qualitative methods in rapid turn-around health services research. Health Services Research & Development Cyberseminar, Spotlight on Women’s Health.

[64] Beebe J (2014). Rapid Qualitative Inquiry: A Field Guide to Team-Based Assessment.

[65] Lewinski AA, Crowley MJ, Miller C (2021). Applied rapid qualitative analysis to develop a contextually appropriate intervention and increase the likelihood of uptake. Med Care.

[66] Vindrola-Padros C, Johnson GA (2020). Rapid techniques in qualitative research: a critical review of the literature. Qual Health Res.

[67] Schexnayder J, Perry KR, Sheahan K (2023). Team-based qualitative rapid analysis: approach and considerations for conducting developmental formative evaluation for intervention design. Qual Health Res.

[68] Finlay L, Finlay L, Gough B (2008). Reflexivity: A Practical Guide for Researchers in Health and Social Sciences.

[69] Ibrahim N The effectiveness of a persuasive mobile app to influence habit change.

[70] Psihogios AM, El-Khatib A, Matos K (2025). Human-centered design of a personalized digital health intervention to improve oral chemotherapy adherence in adolescents and young adults with hematologic cancers. Pediatr Blood Cancer.

[71] Carrera Diaz K, Yau J, Iverson E (2025). Human-centered design approach to building a transition readiness mHealth intervention for early adolescents. J Pediatr Psychol.

[72] Newman SD, Andrews JO, Magwood GS, Jenkins C, Cox MJ, Williamson DC (2011). Community advisory boards in community-based participatory research: a synthesis of best processes. Prev Chronic Dis.

[73] Khowaja K, Al-Thani D (2020). New Checklist for the Heuristic Evaluation of mHealth Apps (HE4EH): development and usability study. JMIR Mhealth Uhealth.

[74] Georgsson M, Staggers N, Årsand E, Kushniruk A (2019). Employing a user-centered cognitive walkthrough to evaluate a mHealth diabetes self-management application: a case study and beginning method validation. J Biomed Inform.

[75] Sayegh CS, Iverson E, MacDonell KK, Wu S, Belzer M (2024). Youth perspectives on mobile health adherence interventions: a qualitative study guided by the supportive accountability model. Patient Educ Couns.

[76] Sayegh CS (2026). Adaptive cell phone support prototyping study. Open Science Framework.

