# Supplementary material for: Feasibility, Usability, and Acceptability of an Adaptive Mobile Health Medication Adherence Intervention for Youth: Iterative Mixed Methods Human-Centered Design Study
Source: JMIR Form Res. 2026 Aug 5;10:e87124. doi: 10.2196/87124 (PMC13441118; doi:10.2196/87124)
Supplement: Supplementary file 1 [file formative-v10-e87124-s001.docx]

Multimedia Appendix 1. Brief history of Cell Phone Support intervention and this team’s rationale for re-design.

Our research team identified that Cell Phone Support, a phone call-based problem-solving intervention, had high potential scalability due to its structure of offering remote, time-limited support from para-professional coaches. Its emphasis on supportive accountability and problem-solving also made Cell Phone Support a promising candidate for adaptation to a transdiagnostic behavior-focused model (and therefore, increased scalability), as these change mechanisms appear to be efficacious across different diagnoses. To demonstrate that Cell Phone Support could extend beyond the original population of AYA living with HIV, we piloted Cell Phone Support with AYA with heart, kidney, and liver transplants, observing medium-to-large improvements in self-reported and biochemically-confirmed adherence. However, only 53% of eligible AYA (9 out of 17) approached about the study enrolled. Moreover, although participants reported that coaching was helpful, they indicated most AYA find phone calls unappealing, which was consistent with literature on youth communication preferences.

At this stage (2020), we convened a Community Advisory Board (CAB) focused on digital health supports for youth with chronic illness, at our hospital. We reviewed the literature on scalability and discussed these concepts with the CAB over several years. We reflected on how acceptability may impact the number of people willing to use an intervention, impacting scalability even if technically an intervention is highly scalable. Therefore, based on participant feedback about the low acceptability of phone calls, our team sought to assess whether delivering Cell Phone Support via text messages could better match AYA communication preferences, while maintaining efficacy. In 2020, we conducted a three-arm randomized trial with a sample of AYA with various chronic health conditions (i.e., heart, kidney, and liver transplants, sickle cell disease, type 2 diabetes) contrasting Cell Phone Support via phone calls, Cell Phone Support via text messages, or automated text message reminders. In this study, enrollment rates improved to 68% of eligible participants (34 out of 50). Although this pilot study was not powered for null-hypothesis statistical testing, Cell Phone Support demonstrated a larger increase in adherence compared to control regardless of whether coaching was delivered by phone or text message (e.g., Cell Phone Support participants reported raising their adherence between 8.59% to 25.80% more than control participants on a scale assessing how often they took their medications out of 100%). However, quantitative and qualitative data suggested that Cell Phone Support by phone call may have improved adherence more than Cell Phone Support by text message. In qualitative feedback, AYA described a tension between phone calls likely being more effective, but text messages being more acceptable. Participants also highlighted how the intensity of Cell Phone Support coaching (whether through calls or texts) could be perceived as potentially annoying, intrusive, unnecessary, or uncomfortable, causing eligible youth to decline participation. They recommended re-designing Cell Phone Support to provide more choice over coaching frequency and communication modality to boost acceptability and engage skeptical youth in the intervention by giving them engagement options.

We discussed this feedback in CAB meetings and mutually concluded to apply for formative research funding to re-design Cell Phone Support using a “foot-in-the-door” technique, which is a persuasion strategy where a small, initial request is followed by a larger request, increasing the likelihood of compliance with the larger request. We collaborated on a plan to reformat Cell Phone Support into an adaptive intervention, starting out with automated adherence coaching that might be more easily accepted by AYA, and then sequentially offering the option to engage with a human coach if the lower-intensity format was insufficient for achieving consistent medication adherence. We hypothesized that this adaptive format could enhance scalability by increasing acceptability, and also by further reducing intervention workforce requirements, as the intensity of human coaching would vary based on each participants’ preferences and response to treatment, instead of following a one-size-fits-all model. We collaborated on several grant proposals and obtained sufficient funding to begin prototyping and user testing our idea for Adaptive Cell Phone Support.

Cell Phone Support Publications to Learn More (In Chronological Order)

Puccio JA, Belzer M, Olson J, Martinez M, Salata C, Tucker D, Tanaka D. The use of cell phone reminder calls for assisting HIV-infected adolescents and young adults to adhere to highly active antiretroviral therapy: a pilot study. AIDS patient care and STDs. 2006 Jun;20(6):438-44.

Belzer ME, Naar-King S, Olson J, et al. The use of cell phone support for non-adherent HIV-infected youth and young adults: an initial randomized and controlled intervention trial. *AIDS and Behavior.* 2014 Apr;18(4). DOI: 10.1007/s10461-013-0661-3. PMID: [24271347](https://pubmed.ncbi.nlm.nih.gov/24271347/)

Belzer ME, Kolmodin MacDonell K, Clark LF, Huang J, Olson J, Kahana SY, Naar S, Sarr M, Thornton S, Adolescent Medicine Trials Network for HIV/AIDS Interventions. Acceptability and feasibility of a cell phone support intervention for youth living with HIV with nonadherence to antiretroviral therapy. AIDS patient care and STDs. 2015 Jun;29(6):338-45.

Sayegh CS, MacDonell KK, Clark LF, et al. The impact of cell phone support on psychosocial outcomes for youth living with HIV nonadherent to antiretroviral therapy. *AIDS and Behavior.* 2018;22(10). DOI: 10.1007/s10461-018-2192-4. PMID: [29948339](https://pubmed.ncbi.nlm.nih.gov/29948339/)

Sayegh CS, Szmuszkovicz JR, Menteer J, Sherer S, Thomas D, Lestz R, Belzer M. Cell phone support to improve medication adherence among solid organ transplant recipients. *Pediatric Transplantation*. 2018;22(6). DOI: 10.1111/petr.13235. PMID: [29920879](https://pubmed.ncbi.nlm.nih.gov/29920879/)

Sayegh CS, MacDonell KK, Iverson E, et al. Randomized pilot trial of cell phone support to improve medication adherence among adolescents and young adults with chronic health conditions. *BMC digital health.* 2024;2(1). DOI: 10.1186/s44247-024-00069-w. PMID: [39211575](https://pubmed.ncbi.nlm.nih.gov/39211575/)

Sayegh CS, Iverson E, MacDonell KK, Wu S, Belzer M. Youth perspectives on mobile health adherence interventions: A qualitative study guided by the supportive accountability model. *Patient education and counseling*. 2024;119. DOI:10.1016/j.pec.2023.108079. PMID: 37995490
