# Supplementary material for: Feasibility, Usability, and Acceptability of an Adaptive Mobile Health Medication Adherence Intervention for Youth: Iterative Mixed Methods Human-Centered Design Study
Source: JMIR Form Res. 2026 Aug 5;10:e87124. doi: 10.2196/87124 (PMC13441118; doi:10.2196/87124)
Supplement: Supplementary file 2 [file formative-v10-e87124-s002.docx]

Multimedia Appendix 2. Community advisory board agendas and resulting action items.

| Date | Agenda | Action Items |
| --- | --- | --- |
| July 2023 | 1) Ice Breaker  2) Review awarded grant proposal, research aims.  3) Orient CAB to CIAS platform, capabilities, other researchers’ uses, and goals.  4) Brainstorm structure, features, content, and visual style. Discuss and draw/type on shared virtual whiteboard, prioritize barriers and solutions for CIAS problem-solving bank. Create a visual flowchart to guide prototype.  4) Set agenda items for next meeting. | 1) Researchers tasked with building CIAS prototype focused on barriers of “forgetting” and “medication issues (Unpleasant side effects, Don’t like the taste, Trouble swallowing pills)” and solutions (alarms, store medication visibility, notes, ask medical team for help, coping with distress, therapy, researching reputable online resources).  2) Use narrator function and personalize with piping in first name of participant  3) Maximum of 5 webpages to click through for single use of program. |
| September 2023 | 1) Ice Breaker  2) Demo automated coaching prototype on CIAS (share screen). CAB members direct research what to click and type to explore the prototype.  3) Facilitate discussion of what’s working well, what is missing, what’s not working. Discuss usability, style/tone, and content.  4) Identify design changes.  5) Consider whether ready for 1^st^ cohort of user testers.  6) Set agenda items for next meeting. | 1) Research team tasked with adding more options to barrier bank and solution bank ahead of next meeting based on today’s discussion.  2) Consensus to refine design further ahead of enrolling user testers, and meet again to test out whether planned changes are sufficient. |
| November 2023 | 1) Ice Breaker  2) Provide personalized links to CAB members to test CIAS.  3) Facilitate discussion of what’s working well, what is missing, what’s not working.  4) Identify design changes.  5) Consider whether ready for 1^st^ cohort of user testers.  6) Set agenda items for next meeting. | 1) Minor improvements should be made by researchers based on today’s conversation  1) Fix usability problems (especially with registering and launching first use, also when using by mobile device versus laptop, some visibility issues). Requirements for name piping are too onerous, adds to much friction for user.  2) Identified off-putting wording that should be revised in several places.  3) Make human support option clearer earlier.  4) Consensus to enroll 1^st^ cohort of user testers after researchers make above revisions, without needing another meeting first. |
| March 2024 | 1) Ice Breaker  2) Update on recruitment  3) Discuss aims and strategy for federal grant proposal to pilot test intervention after user testing complete.  4) Draft letter of support from CAB. | 1) Researchers will continue recruitment efforts.  2) Researchers will submit federal grant proposal for October deadline. |
| May 2024 | 1) Ice Breaker  2) Present summary of Cohort 1 user feedback.  3) Facilitate discussion of what’s important from this feedback? What changes should we make to the prototype?  4) Discuss challenges recruiting young men. Brainstorm strategies.  5) Set agenda items for next meeting. | 1) Researchers tasked with removing narrator, adding short video to introduce human coaching better.  2) Concerned about representativeness of sample (all female, skew older/college students). Before making too many adjustments to prototype, put effort into recruiting more user testers for 2^nd^ cohort including young men and younger teens.  3) Change recruitment materials to emphasize how enrolling can be an altruistic way to help others via what is being learned and created, not emphasizing how people might enroll because they are the ones needing help.  4) Consensus to enroll 2^nd^ cohort of user testers after researchers make above revisions, without needing another meeting first. |
| December 2024 | 1) Ice Breaker  2) Present summary of Cohort 2 user feedback.  3) Facilitate discussion of what’s important from this feedback? What changes should we make to the prototype?  5) Set agenda items for next meeting. | 1) Researchers tasked with refining prototype in several ways: replace “app” term due to confusion, add more orienting information, refine wording in several places, add 4 barriers, integrate resources into solution bank instead of separate text message, add coach outreach texts/calls, encourage calls over texts without forcing it.  3) Consensus to enroll 3^rd^ cohort of user testers after researchers make above revisions, without needing another meeting first. |
| March 2025 | 1) Ice Breaker  2) Present summary of Cohort 3 user feedback.  3) Facilitate discussion of what’s important from this feedback? What changes should we make to the prototype?  4) Discuss ways for CAB members to be involved in dissemination efforts.  5) Set agenda items for next meeting. | 1) Researchers tasked with refining prototype in several ways: change name intervention name because “CPS” is triggering/misunderstood due to associate with foster care, improve first impressions, add variety to language and images, add two barriers, offer short coach phone call to everyone the first week, make sure coaches are referencing what participants input into CIAS in their conversations.  2) Researchers will continue efforts at obtaining federal funding for pilot trial. Plan re-submit if needed, and consult CAB further.  3) Four CAB members will join manuscript writing team. Others are not interested. |
