# Supplementary material for: Feasibility, Usability, and Acceptability of an Adaptive Mobile Health Medication Adherence Intervention for Youth: Iterative Mixed Methods Human-Centered Design Study
Source: JMIR Form Res. 2026 Aug 5;10:e87124. doi: 10.2196/87124 (PMC13441118; doi:10.2196/87124)
Supplement: Supplementary file 3 [file formative-v10-e87124-s004.docx]

Multimedia Appendix 4. Think-aloud guide and semistructured interview guides.

User-Testing Qualitative Guide

1) Check that person completed consents.
2) Distribute initial REDCap surveys (Demographics, Theoretical Framework of Acceptability Questionnaire, 5 Stars, and Visual Analogue Scale).
   - Send survey link in the chat
   - Open Record Status Dashboard, and check that there are green check marks in Surveys, Medication Adherence, TFA, and Before Stars. Thank them.
3) Interview 1:
   - Before the 3-week trial period, schedule a one-hour user-testing session with the participant to assess their immediate reactions to the study intervention. Interviewer statements and questions are bolded.
   - If you were in charge of the hospital, how would you change things?
   - Thank you so much for taking the time to try out the phone-based program for supporting youth in taking their medications consistently. Starting today, you’ll have a three-week trial period to test out this program. We’ll meet again in three weeks to go over your feedback, but today, I’d like to show you the program and learn what you think about it. We’re going to audio-record today’s session so I can remember what happens. Later, we’ll summarize the main points from the recording and delete it to protect your privacy.
   - Help participant load the intervention on their phone.
   - Now, I’m going to ask you to check out the program and try out different parts of it. I would appreciate it if you can talk out loud about anything you are thinking and doing, so I can understand what it’s like for you. I can show you an example of talking out loud while doing something. Ok, here I have this magazine. I can see on the cover that it is about [demonstrate with real object], and I like that it has lots of colors and photography. It makes me want to open it up and check it out. Ok I’m opening it up to the first page—alright I see an ad. This doesn’t seem that relevant to me, let’s turn the page. Ok, here’s the table of contents. I’m going to scan through and see if any of the articles seem interesting. Yeah this one seems, cool, I’m going to turn to page 10. Alright, so I’m seeing a lot of tiny words here and thinking I’m not sure I have time to read this right now. I’ll fold down the edge to go back to it later… So that’s an example of me talking out loud about my experience doing something. I’m going to ask you to do the same as you check out our program. There’s no right or wrong answers! I just want to hear what’s going on for you in your own words.
   - Ok, start out by opening the program and just go from there.
   - When the participant stops narrating or interacting with the program, or after 10 minutes, ask them to try the following activities.
   - Thanks, now I’m going to have you respond to a medication check-in. The program will send you a text, asking to check in about your recent medication taking. Please respond to the check in and think out loud again, sharing with me what you are thinking, doing, and experiencing.
   - When the participant stops narrating or interacting with the program, or after 5 minutes, ask them to try the following activities.
   - Great, now I’m going to have you try the problem-solving activity. The program will help you identify barriers to taking medication consistently and help you identify solutions that could help. Please try this out and think out loud again, sharing with me what you are thinking, doing, and experiencing.
   - When the participant stops narrating or interacting with the program, or after 5 minutes, ask them to try the following activities.
   - Awesome, now I’m going to have you try accessing a community resource. The program has a library of resources that can help youth take their medication consistently. Check this part out and think out loud again, sharing with me what you are thinking, doing, and experiencing.
   - When the participant stops narrating or interacting with the program, or after 5 minutes, ask them to try the following activities.
   - Alright, now I’m going to have you try asking for help from a coach. The program has a way to ask for support from a person who is trained to help youth take their medication consistently. Please try asking for a coach and think out loud again, sharing with me what you are thinking, doing, and experiencing.
   - When the participant stops narrating or interacting with the program, or after 5 minutes, ask them to try the following activities.
   - Ok, feel free to go back to any part to check it out further and talk through what you’re thinking.
   - Now I would like to ask you a few questions.
     i. First, this question is a little silly and takes imagination but could help us warm up. If this intervention was an animal, what would it be and why?
     ii. Overall, what do you think of the program?
     iii. How easy or hard was it to use the program? Please explain.
     iv. How helpful or not do you find the program? Please explain.
     v. How did you feel while trying the program?
     vi. How well did you understand how the program was supposed to work?
     vii. How much would using the program interfere with your life?
     viii. How much do you think you’ll use this program over the next three weeks? Please explain.
     ix. Do you have any recommendations for improving the program?
     x. What do you think other people your age would think of the program?

- Thank you so much for your time today. We’d appreciate it if you could also now fill out these surveys via REDCap (mHealth App Usability Questionnaire, t stars, Theoretical Framework of Acceptability Questionnaire). Also, let’s schedule your follow-up interview.
- REDCap
  - Click the blank dot under “Stars” in “Event 1”.
  - Click Survey Options, then Open Survey, then this will open a new window, copy the URL, and send it to them in the chat. Close the window on your computer and select Leave without Saving.
  - Open Record Status Dashboard, and check that there are green check marks in Stars, Mauq, and TFA Measure After Trying Intervention. Thank them.
  - Suggest they save the phone number CIAS will be using in their contacts.

4) Interview 2:

- After the 3-week trial period, schedule a one-hour user-testing session with the participant to assess their immediate reactions to the intervention.
- REDCap
  - Click the blank dot under “Medication Adherence in” in “3 Week Usability”.
  - Click Survey Options, then Open Survey, then this will open a new window, copy the URL, and send it to them in the chat. Close the window on your computer and select Leave without Saving.
  - Open Record Status Dashboard, and check that there are green check marks in Medication Adherence, Stars, Mauq, and TFA Measure After Trying Intervention. Thank them.
- As a reminder, I’ll be recording the interview to help me remember what you say. Press record.
- Thank you so much for trying the program out over the past few weeks. I would love to ask you some questions about your experience.
- First, which of these pictures is most similar to the intervention in your experience? Why do you say so?

[Show picture sheet selected from google images search by researchers]

- Overall, what do you think of the program?
- If you ever interacted with the coach, what was that like for you?
- When or how do you think the coach should reach out to people using the phone tool?
- How easy or hard was it to use the program? Please explain.
- How helpful or not do you find the program? Please explain.
- How did you feel while trying the program?
- How well did you understand how the program was supposed to work?
- How much would using the program interfere with your life?
- Do you have any recommendations for improving the program? Any ideas for a good name for this program?
- What do you think other people your age would think of the program?
- Although the 3 week trial period is ending now and we aren’t offering the tool for longer yet, would you have wanted to continue using the tool after today? Why or why not?
