# Supplementary material for: Feasibility, Usability, and Acceptability of an Adaptive Mobile Health Medication Adherence Intervention for Youth: Iterative Mixed Methods Human-Centered Design Study
Source: JMIR Form Res. 2026 Aug 5;10:e87124. doi: 10.2196/87124 (PMC13441118; doi:10.2196/87124)
Supplement: Supplementary file 4 [file formative-v10-e87124-s005.docx]

Multimedia Appendix 5. Rapid assessment template.

Prepared By: __________	Participant ID:

Instructions: Please summarize any participant responses relevant to those domains. Note timestamps for any particularly illustrative quotations. Do not exceed a total of 2 pages.

Think-Aloud Comments:

Description of Their Own Medication Adherence:

Personal Views of Current Version of Intervention (Based on the 1^st^ Try):

The Check In:

Problem Solving Module:

The Coaching:

The Resource Library:

Other:

Personal Views of Current Version of Intervention (Based on the 3-week Trial Period):

The Check In:

Problem Solving Module:

The Coaching:

The Resource Library:

Other:

Recommendations for Improving the Intervention:

The Check In:

Problem Solving Module:

The Coaching:

The Resource Library:

Other:

How Others Might Perceive Intervention, How It Might Work If Implemented at The Hospital:
