# Supplementary material for: Feasibility, Usability, and Acceptability of an Adaptive Mobile Health Medication Adherence Intervention for Youth: Iterative Mixed Methods Human-Centered Design Study
Source: JMIR Form Res. 2026 Aug 5;10:e87124. doi: 10.2196/87124 (PMC13441118; doi:10.2196/87124)
Supplement: Supplementary file 5 [file formative-v10-e87124-s006.docx]

Multimedia Appendix 6. Usability and acceptability item-level ratings.

|  | Cohort 1  *n* = 6 | Cohort 2  *n* = 6 | Cohort 3  *n* = 10 |
| --- | --- | --- | --- |
|  | *M (SD)* | *M (SD)* | *M (SD)* |
| MAUQ |  |  |  |
| Easy to use, T1 | 6.67 (0.82) | 7.00 (0.00) | 6.70 (0.48) |
| Easy to use, T2 | 6.50 (0.84) | 6.50 (0.84) | 7.00 (0.00) |
| Easy to learn, T1 | 6.50 (0.84) | 7.00 (0.00) | 6.80 (0.42) |
| Easy to learn, T2 | 6.67 (0.82) | 7.00 (0.00) | 7.00 (0.00) |
| Like the interface, T1 | 6.17 (0.75) | 6.67 (0.82) | 6.30 (1.06) |
| Like the interface, T2 | 6.33 (0.52) | 6.17 (0.98) | 5.90 (1.10) |
| Information easy to find, T1 | 6.67 (0.52) | 6.67 (0.82) | 6.70 (0.48) |
| Information easy to find, T2 | 6.33 (0.82) | 6.00 (1.27) | 6.40 (0.70) |
| Comfortable using in social settings, T1 | 6.33 (0.82) | 6.17 (1.17) | 6.50 (0.71) |
| Comfortable using in social settings, T2 | 5.83 (1.17) | 6.00 (1.27) | 6.56 (0.73)^a^ |
| Fitting amount of time required, T1 | 6.33 (1.03) | 6.83 (0.41) | 6.60 (0.52) |
| Fitting amount of time required, T2 | 6.17 (1.33) | 6.17 (1.33) | 6.56 (0.88)^a^ |
| Would use again, T1 | 6.50 (0.84) | 6.17 (1.32) | 6.30 (1.89) |
| Would use again, T2 | 6.67 (0.82) | 5.67 (2.42) | 5.90 (1.52) |
| Overall satisfied, T1 | 6.67 (0.52) | 6.67 (0.82) | 6.60 (0.52) |
| Overall satisfied, T2 | 6.67 (0.82) | 6.83 (0.41) | 5.60 (1.27) |
| Useful for my health, T1 | 6.83 (0.41) | 6.83 (0.41) | 6.50 (0.53) |
| Useful for my health, T2 | 6.67 (0.82) | 6.67 (0.82) | 5.90 (1.85) |
| Improved access to services, T1 | 6.67 (0.52) | 5.00 (2.45) | 5.50 (0.97) |
| Improved access to services, T2 | 6.33 (0.82) | 5.83(1.84) | 5.30 (1.42) |
| Helped me manage my health, T1 | 6.67 (0.52) | 6.00 (1.26) | 5.80 (0.79) |
| Helped me manage my health, T2 | 6.50 (0.84) | 6.50 (0.84) | 5.30 (1.25) |
| Convenient to communicate with provider, T1 | 6.17 (0.98) | 5.50 (1.52) | 5.50 (1.43) |
| Convenient to communicate with provider, T2 | 6.50 (0.84) | 5.50 (1.98) | 5.20 (1.81) |
| Opportunities to interact with provider, T1 | 6.50 (0.84) | 5.50 (1.52) | 5.20 (1.99) |
| Opportunities to interact with provider, T2 | 6.50 (0.84) | 4.83 (1.94) | 5.10 (1.91) |
| Confident information would be received, T1 | 6.50 (0.55) | 5.17 (1.83) | 5.60 (1.51) |
| Confident information would be received, T2 | 6.00 (0.89) | 5.33 (1.86) | 6.40 (0.84) |
| Comfortable communicating with provider, T1 | 6.17 (1.33) | 6.00 (1.67) | 5.70 (1.89) |
| Comfortable communicating with provider, T2 | 6.67 (0.82) | 5.67 (1.51) | 6.20 (1.03) |
| TFA |  |  |  |
| TFA Affective attitude, T0 | 4.17 (2.33) | 3.67 (0.82) | 4.10 (0.74) |
| TFA Affective attitude. T1 | 4.50 (0.55) | 4.50 (0.55) | 4.20 (0.42) |
| TFA Affective attitude. T2 | 4.00 (0.89) | 4.17 (0.75) | 4.30 (0.68) |
| TFA Burden^a^, T0 | 2.67 (1.21 | 3.17 (1.17) | 2.80 (1.14) |
| TFA Burden^a^, T1 | 2.17 (1.17) | 2.50 (1.38) | 1.90 (0.57) |
| TFA Burden^a^, T2 | 2.17 (1.17) | 2.83 (1.72) | 2.00 (1.16) |
| TFA Perceived Effectiveness, T0 | 3.67 (0.82) | 3.17 (1.47) | 3.60 (1.17) |
| TFA Perceived Effectiveness, T1 | 3.50 (0.84) | 3.67 (0.82) | 3.40 (0.70) |
| TFA Perceived Effectiveness, T2 | 4.00 (0.63) | 4.33 (0.82) | 4.00 (0.82) |
| TFA Ethicality, T0 | 4.17 (0.98) | 3.50 (1.52) | 4.20 (0.63) |
| TFA Ethicality, T1 | 4.33 (1.03) | 3.83 (0.75) | 4.20 (0.63) |
| TFA Ethicality, T2 | 4.50 (0.55) | 4.17 (0.99) | 4.50 (0.53) |
| TFA Opportunity Costs^a^, T0 | 3.33 (1.03) | 2.33 (1.51) | 2.30 (0.48) |
| TFA Opportunity Costs^a^, T1 | 3.33 (1.21) | 2.67 (1.51) | 2.30 (0.95) |
| TFA Opportunity Costs^a^, T2 | 2.33 (1.37) | 2.17 (1.47) | 2.10 (1.00) |
| TFA Self-efficacy, T0 | 4.17 (0.41) | 2.67 (1.37) | 4.00 (0.67) |
| TFA Self-efficacy, T1 | 4.50 (0.55) | 4.33 (0.52) | 4.30 (0.48) |
| TFA Self-efficacy, T2 | 4.33 (0.52) | 4.50 (0.55) | 4.30 (0.82) |
| TFA Intervention Coherence, T0 | 4.33 (0.52) | 4.00 (0.89) | 3.90 (0.88) |
| TFA Intervention Coherence, T1 | 4.67 (0.52) | 4.50 (0.55) | 4.30 (0.48) |
| TFA Intervention Coherence, T2 | 4.33 (0.52) | 4.67 (0.52) | 4.10 (0.99) |

^a^One participant skipped two items, which contributed to a missing Ease of Use subscale for that participant.
